# Generation of Recombinant Monoclonal Antibodies from Immunised Mice and Rabbits via Flow Cytometry and Sorting of Antigen-Specific IgG^+^ Memory B Cells

**DOI:** 10.1371/journal.pone.0152282

**Published:** 2016-03-29

**Authors:** Dale. O Starkie, Joanne E. Compson, Stephen Rapecki, Daniel J. Lightwood

**Affiliations:** UCB Pharma, 216 Bath Road, Slough, Berkshire, SL1 3WE, United Kingdom; King's College London, UNITED KINGDOM

## Abstract

Single B cell screening strategies, which avoid both hybridoma fusion and combinatorial display, have emerged as important technologies for efficiently sampling the natural antibody repertoire of immunized animals and humans. Having access to a range of methods to interrogate different B cell subsets provides an attractive option to ensure large and diverse panels of high quality antibody are produced. The generation of multiple antibodies and having the ability to find rare B cell clones producing IgG with unique and desirable characteristics facilitates the identification of fit-for-purpose molecules that can be developed into therapeutic agents or research reagents. Here, we describe a multi-parameter flow cytometry single-cell sorting technique for the generation of antigen-specific recombinant monoclonal antibodies from single IgG^+^ memory B cells. Both mouse splenocytes and rabbit PBMC from immunised animals were used as a source of B cells. Reagents staining both B cells and other unwanted cell types enabled efficient identification of class-switched IgG^+^ memory B cells. Concurrent staining with antigen labelled separately with two spectrally-distinct fluorophores enabled antigen-specific B cells to be identified, i.e. those which bind to both antigen conjugates (double-positive). These cells were then typically sorted at one cell per well using FACS directly into a 96-well plate containing reverse transcriptase reaction mix. Following production of cDNA, PCR was performed to amplify cognate heavy and light chain variable region genes and generate transcriptionally-active PCR (TAP) fragments. These linear expression cassettes were then used directly in a mammalian cell transfection to generate recombinant antibody for further testing. We were able to successfully generate antigen-specific recombinant antibodies from both the rabbit and mouse IgG^+^ memory B cell subset within one week. This included the generation of an anti-TNFR2 blocking antibody from mice with an affinity of 90 pM.

## Introduction

Since Kohler and Milstein first described a method for the generation of monoclonal antibodies (mAbs) via their hybridoma technology in 1975 [1], mAbs have become both essential research reagents and highly successful therapeutic molecules. In 2014 five out of the top ten best selling drugs were antibody-based including Humira™, the highest seller. At the time of writing this, a total of 43 antibodies have received FDA approval for use as therapeutics and many more are currently in development [2]. As disease targets become more challenging to modulate through antibody intervention due to their high sequence conservation across species (making immunisation difficult), restricted anatomical location (e.g. CNS), difficulty in purifying a soluble form (e.g. GPCRs) and the need to sometimes target disease state-specific transient or unstable conformations, it is preferable to have access to a number of antibody discovery technologies that allow for a diverse panel of molecules to be generated and tested. This includes both immunisation-dependent and independent methods. Such a strategy increases the chances of discovering those antibodies with highly desirable characteristics, providing the best chance of delivering effective antibody treatments for patients suffering with serious disease.

Although the hybridoma method has revolutionised the use of monoclonal antibodies, the technology is relatively inefficient (5 × 10^−6^ efficiency with conventional PEG fusion) due to its reliance on a fusion event [3]. As a result, many B cells do not get sampled and the potential diversity in an immune repertoire is consequently not interrgoated. Display methodologies, such as phage and yeast display, have also been widely used as a technology for producing monoclonal antibodies [4,5]. However, the random combination of antibody variable region genes which occurs during library construction results in the loss of natural cognate heavy and light chain pairings that are evolved and selected for *in vivo* during an immune response [6,7]. As a result of this random pairing, antibodies from naïve antibody libraries typically require *in vitro* maturation to impart increased affinity and stability prior to progression as a therapeutic molecule.

In recent years, there has been an emergence of a number of single-B cell technologies that allow the direct sampling of the immune repertoire (reviewed by Tiller) [8]. These platforms retain the natural heavy and light chain pairing and avoid the inefficient hybridoma fusion step, thereby enabling efficient mining of the immune B cell population. This facilitates the discovery of rare antibodies that may possess unique highly desirable properties as well as the generation of large and diverse panels of antibodies. The preservation of the natural heavy and light chain pairings during cloning of antibody genes favours the generation of recombinant antibodies with an attractive affinity, specificity and stability profile. Of note are techniques that sample IgG-secreting cells such as plasma cells, including the fluorescent foci method [9] and a number of microengraved array technologies [10–16]. Despite the attraction of sampling the plasma cell repertoire from niches such as the bone marrow, the methods for single cell isolation are currently reliant on manual micromanipulation and are therefore low throughput.

Flow cytometry has been used to isolate single plasmablasts from blood of human donors taken 7 days following an immunization, vaccination or infection. Plasmablasts appear transiently in the periphery during this time and provide an enriched population of antigen-specific B cells from which to select [6–8,17,18]. However, sorting does not incorporate an antigen-binding step and despite the enriched population, recovery of antigen specific recombinant antibodies from these plasmablasts can be as low as 10% of sorted cells [7].

Other than work by Manz *et al*. [19] and more recently Carroll and Al-Rubeai [20] and Taddeo *et al*. [21], flow cytometry has not been applied routinely to the identification of antigen-reactive IgG-secreting cells. This is mainly due to the lack of surface immunoglobulin, precluding the option of staining specific cells with a target antigen. However, FACS has been successfully employed in the identification of antigen-specific memory B cells, expressing surface IgG as part of the B cell receptor [22–31]. Weitkamp *et al*. (2003) [30] used fluorescent virus-like particles (VLPs) to identify and sort single rotavirus-specific human B cells into single wells of a 96-well plate before culturing to induce antibody secretion. Amanna and Slifka [22] devised a FACS method for the identification and quantitation of tetanus and diphtheria antigen-specific memory B cells in clinical samples. However, they did not extend this to single cell sorting and further analysis. di Niro *et al*. [31] described rotavirus-specific B cell sorting immediately followed by single cell RT-PCR recovery of cognate heavy and light chain variable regions and subsequent recombinant antibody production. More recently, Franz *et al*. [23] described the use of labelled tetrameric tetanus toxin C-fragment to identify and sort rare memory B cells from healthy volunteers. This work, and the vast majority of other published data describing single cell sorting of antigen-specific B cells and plasmablasts, appears to be largely restricted to humans where large samples of blood can be easily taken and prepared for FACS sorting. The human system is also well furnished with an extensive array of highly characterised antibody reagents for identifying cell subsets. There are much fewer examples in the literature of using non-human animal species, that allow for immunization with a broad spectrum of molecules, being used as a source of target-specific B cells for subsequent single cell sorting using flow cytometry. Lalor *et al*. [32] were one of the first to describe a multiparameter flow cytometric sorting protocol in rodents followed by variable region gene recovery and analysis. However, this was performed using the hapten (4-hydroxy-3-nitrophenyl) acetyl (NP) in C57BL/6 mice followed by focused analysis of the dominant V186.2 heavy chain variable region gene only. Despite this early development, there have been few published examples of flow cytometric sorting of antigen-specific memory B cells from immunized animals for the purpose of monoclonal antibody discovery. Townsend *et al*. [29] described the identification of rare antigen-specific murine B cells through the use of antigen conjugated with two different fluorophores (FITC and PE). The two-colour system provided increased accuracy in subset identification and minimised the non-specific staining of other cells. Again, their work did not cover single cell sorting and variable region gene recovery. Tiller *et al*. [33] described the recovery of Ig genes from single sorted mouse B cells but in this case the FACS protocol did not incorporate an antigen-staining step and it can be assumed that such an approach would be an inefficient method for recovery of antigen-specific monoclonal antibodies from the IgG^+^ memory B cell pool.

**Fig 1:**
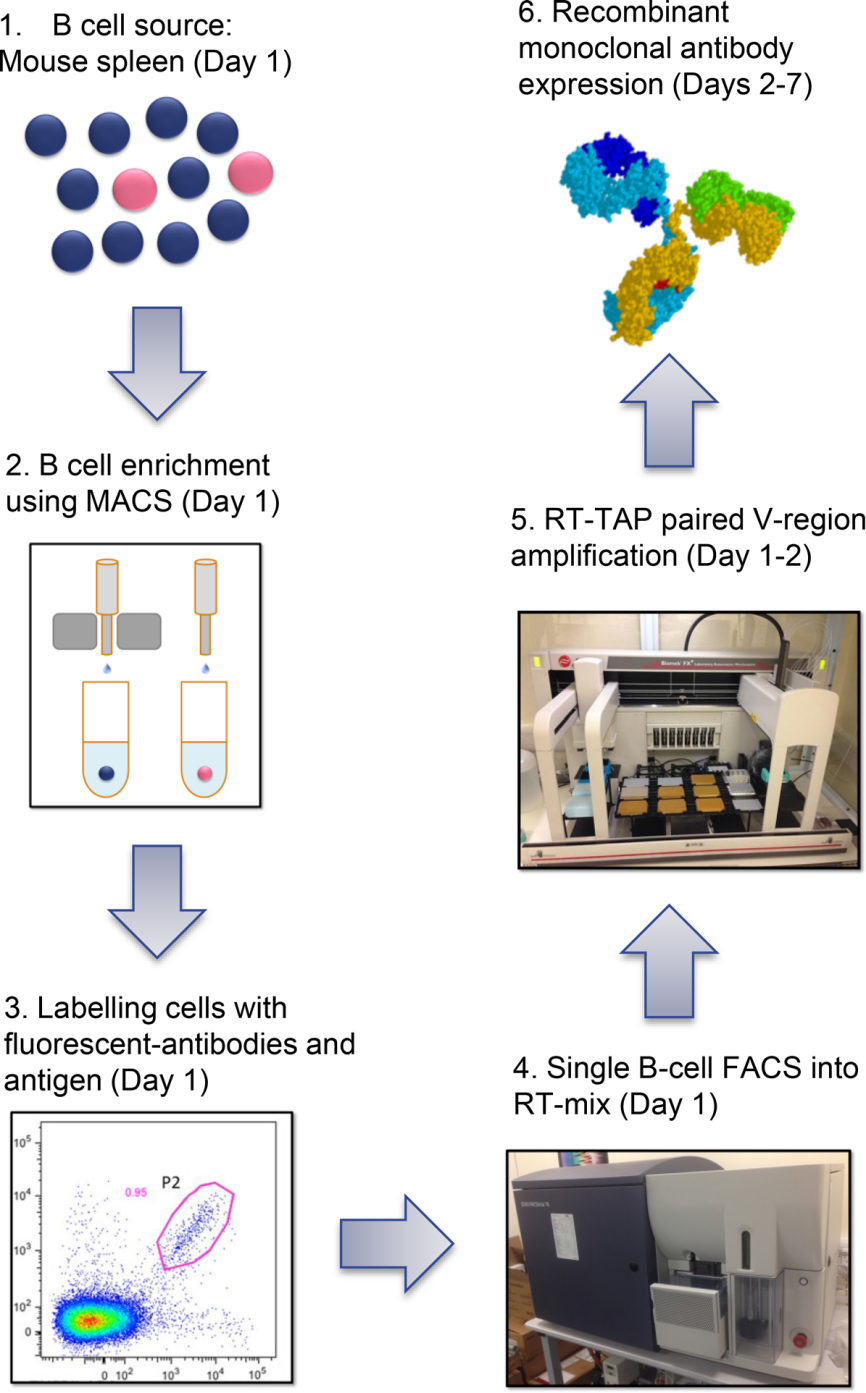
Schematic representation of the single-B cell sorting protocol used for antibody discovery from immunised mice. The following steps were undertaken: 1. Mice were immunised and a splenocyte suspension prepared. 2. B cells from the mouse splenocyte preparation were enriched using anti-mouse CD45R microbeads (Miltenyi Biotech) and LS MACS columns (Miltenyi Biotech) according to manufacturers’ instructions. 3. Following enrichment, cells were stained with the following antibodies: rat anti-mouse IgG brilliant violet 421 (BD biosciences), rat anti-mouse IgM PE-Cy7 (Bio legend), rat anti-mouse IgD APC-Cy7 (Bio legend), rat anti-mouse CD19 AF700 (BD biosciences) (2 μg per 108 Cells), and rat anti-mouse CD4, CD8, GR1 and F4/80 FITC (BD biosciences)(dump channel). Human TNFR2 extracellular domain was labelled with PE and APC using Lightning Link PE and APC labelling kits (Innova Bioscience) and added to the cell suspension. 4. FACS was performed on a BD FACS ARIA III with single human TNFR2-specific IgG^+^ B cells being deposited into the well of a 96-well PCR plate. 5. cDNA from single B cells was prepared using Superscript III reverse transcriptase (Invitrogen) primed with oligo (dT). Antibody variable-region genes were then recovered via two rounds of PCR followed by a third round to generate transcriptionally-active PCR (TAP) products in a manner similar to that described in Clargo *et al*.^9^ employing an Aviso Onyx liquid handling robot to facilitate set-up. 6. Heavy and light chain TAP fragments were transiently co-transfected into Expi293 cells using ExpiFectamine (Life Technologies). After 7 days expression, supernatants were harvested for further characterisation.

Here, we describe a multi-parameter flow cytometric single cell sorting technique for the generation of antigen-specific recombinant monoclonal antibodies from immunised mice and rabbits (see Fig 1 for method schematic). In combination with several B cell-surface markers and negative stains (dump channel), two-colour antigen staining enabled efficient identification and sorting of specific B cells from mouse spleen and rabbit PBMC. In the mouse system, the technique included an upfront magnetic activated cell sorting (MACS) step using an anti-CD45R (B220) reagent to enrich for B cells in the spleen of immunised mice. Due to the lack of availability of equivalent reagents in the rabbit system, a MACS step was not included for this example. Mouse B220^+^ B cells were stained with anti-CD19 and anti-IgG antibodies (positive B cell stain). This was combined with staining of unwanted cells using an antibody panel incorporating reactivity to naïve B cells (IgM, IgD), T cells (CD4, CD8), neutrophils (GR-1), macrophages (F4/80) and dead cells (7AAD). The inclusion of antigen that had been conjugated to two different fluorophores (spectrally distinct) into the staining panel allowed for accurate identification of IgG^+^ antigen-specific memory B cells in the flow cytometer. With the absence of a general B cell marker, the rabbit system was restricted to positively staining with antigen (labelled with two fluorophores) and IgG only. However, we were able to include markers for staining unwanted IgM^+^ cells, T cells and dead cells. Following identification of antigen-specific IgG^+^ memory B cells, single cells, or in some cases up to 3 cells (during the rabbit sort), were sorted directly into a well of a 96-well plate containing reverse transcriptase reaction mix. Following production of cDNA, 3 rounds of antibody-specific PCR was performed to amplify heavy and light chain variable region genes and generate a transcriptionally-active PCR (TAP) fragments that could be used directly in a mammalian cell transfection to generate recombinant antibody. This recombinant antibody, produced from HEK-293 cells, was then tested for reactivity with target antigen. We were able to generate antigen-specific antibodies in both the rabbit and mouse system with an overall efficiency of up to 38.5% (antigen-specific recombinant antibodies per sorted single cell). In summary, this method allowed us to generate large panels of antigen-specific recombinant antibodies, derived from IgG^+^ memory B cells, from immunised mice and rabbits within one week. Antibodies produced using this method represent useful reagents in their own right as well as potential starting points from which to engineer and humanise sequences in preparation for clinical development.

## Materials and Methods

### Immunisations

Approval for use of animals for immunisation was provided through the UCB Pharma, UK Animal Welfare and Ethical Review Body (AWERB) and the license was granted by the UK Home Office. At the end of the study the mice were anesthetised with isoflurane, terminal bleeds taken and then sacrificed using a Schedule 1 method in accordance with the Animals Scientific Procedures Act (ASPA). Rabbits were terminally anaesthetised with a Euthatal solution in accordance with Animals Scientific Procedures Act (ASPA).

Five Balb/c mice were immunised subcutaneously with 100 μg of purified human IL-25 (IL7-E) (Peprotech) or human TNFR2 extracellular domain (residues 1–256) (generated in house) per injection with CFA for the first dose and IFA for subsequent boosts. The animals received 2 booster injections at 2 week intervals. Splenocytes and blood for sera analysis were prepared from animals harvested 7 days following the final boost. New Zealand White rabbits were immunised subcutaneously with 500 μg of purified mouse WISP-1-rabbit Fc fusion protein (generated in house) emulsified with CFA for the first dose and IFA for subsequent boosts. The animals received 2 booster injections at 3 week intervals. PBMC for FACS and sera analysis were prepared from whole blood of animals harvested 14 days following the final boost.

### Preparation and staining of mouse B cells for sorting

Mouse splenocytes were harvested using a gentleMACS Dissociator (Miltenyi Biotech). The cells were then pushed through a cell strainer (Fisher Scientific) to generate a single cell suspension before being used for FACS or being frozen in 10% DMSO in FCS. Spleens from all 5 mice were combined prior to FACS. Splenocytes were washed in a 10-fold excess of Hanks balanced salt solution without Mg^2+^ or Ca^2+^ (Life Technologies), 1% FCS (Life Technologies), 25mM HEPES (Sigma), 1mM EDTA (Sigma)(described as buffer solution below). Cells were then blocked using mouse Fc block reagent (Miltenyi Biotech) following the manufacturers’ instructions. Following this, B cells were enriched using anti-mouse CD45R microbeads (Miltenyi Biotech) and LS MACS columns (Miltenyi Biotech) according to manufacturers’ instructions. Following enrichment, cells were resuspended in buffer solution and stained with 1 μg (unless stated otherwise) per 10^8^ cells of the following antibodies: Rat anti-mouse IgG brilliant violet 421 (BD biosciences), rat anti-mouse IgM PE-Cy7 (Bio legend), rat anti-mouse IgD APC-Cy7 (Bio legend), rat anti-mouse CD19 AF700 (BD biosciences) (2 μg per 10^8^ Cells), and rat anti-mouse CD4, CD8, GR1 and F4/80 FITC (BD biosciences) (dump channel). Human IL-25 (Peprotech) and TNFR2 extracellular domain (residues 1–256) (generated in house) were labelled with PE and APC using Lightning Link PE and APC labelling kits (Innova Bioscience) following manufacturers’ guidelines. In each case, the two antigen preparations labelled with both fluorophores were pre-mixed and used at 2 μg total protein per 10^8^ cells. Cells were incubated in the dark for 20 minutes at room temperature and then washed twice in buffer before being finally re-suspended in buffer for FACS sorting.

### Preparation of rabbit B cells for sorting

Rabbit whole blood was mixed 1:1 in PBS and the mixture layered onto Lympholyte mammal (Cedarlane) in a 50 ml sterile Falcon tube (Fisher Scientific). This was centrifuged at 1500 x g for 30 minutes at 5°C. The PBMC layer was removed using a pipette and used for FACS or frozen down in 10% DMSO in FCS. PBMCs were washed in a 10 fold excess of Hanks balanced salt solution without Mg^2+^ or Ca^2+^ (Life Technologies), 1% FCS (Life Technologies), 25mM HEPES (Sigma), 1mM EDTA (Sigma) (described as buffer solution below). Mouse anti rabbit IgM biotin (BD biosciences) was pre incubated with 5 fold excess PE-Cy7 streptavidin, following a 20 minute incubation a 10 fold excess of biotinylated human albumin was added to the mix to remove any free PE-Cy7 streptavidin. Mouse anti-rabbit CD4, CD8 and a Pan T-cell antibody (ABD serotec) were labelled with PerCP using a Lightning Link kit (Innova Biosciences) following the manufacturers’ instructions. Cells were resuspended in buffer and stained with 1 μg per 10^8^ cells with the following antibodies: mouse anti-rabbit IgG PE (Southern biotech), mouse anti rabbit IgM biotin-streptavidin PE-Cy7 complex, mouse anti-rabbit CD4, CD8 and a Pan T-cell antibody PerCP. Mouse WISP-1 (R & D Systems) was labelled with Alexa 647 and Alexa 488 using Microscale labelling kits (Life technologies) following the manufacturer’s instructions. Both labelled antigen preparations were used at 1 μg per 10^8^ cells. Cells were incubated in the dark for 20 minutes at room temperature and then washed twice in buffer before being finally resuspended in buffer for FACS sorting.

### Mouse single B cell sorting

Compensation for the mouse sorting experiments was carried out by labelling mouse splenocytes separately with rat anti-mouse CD45R labelled with the different flourophores that were used in the experiment. Comparison to an unstained control sample provided a clear positive and negative population for each fluorophore colour allowing compensation to be calculated using the BD FACS DIVA software.

FACS sorting was carried out on a BD FACS ARIA III, using a 100 μm sort nozzle at no greater than 2000 events per second. Five minutes prior to running and sorting the samples, 2% v/v 7AAD (BD biosciences) was added to the samples in order to identify and eliminate dead cells. Sorting was controlled using BD FACS DIVA software. Post sort analysis was performed using FlowJo software (Tree Star).

Lymphocytes were identified by size and granularity using FSC-A versus SSC-A. Single lymphocytes were identified and gated using FSC-W versus FSC-A. T-cells, macrophages and neutrophils were identified and eliminated from sorting gates (dump channel). CD19^+^, IgG^+^, IgM^-^, IgD^-^ B cells were identified and gated. Finally a gate was drawn around the double-positive antigen-specific population. All antigen-specific single B cells were sorted at single-cell per well into reverse transcription mix chilled to 4°C in 96-well PCR plates.

### Rabbit single B cell sorting

Compensation for the rabbit sorting was done using anti-mouse compensation beads (BD bioscience) and a series of mouse antibodies covering all the fluorophores used in the experiment and a comparison made to an unstained cell sample. Compensation was again calculated using the BD FACS DIVA software.

FACS sorting was carried out on a BD FACS ARIA III, using a 100 μm sort nozzle at no greater than 2000 events per second. Five minutes prior to running and sorting the samples, 2% v/v 7AAD (BD biosciences) was added to the samples to identify and eliminate dead cells. Sorting was controlled using BD FACS DIVA software. Post sort analysis was performed using FlowJo software (Tree Star). Lymphocytes were identified by size and granularity using FSC-A versus SSC-A. Single lymphocytes were identified and gated using FSC-W versus FSC-A. T-cells were identified and eliminated (dump channel). IgG^+^, IgM^-^ B cells were identified and gated on. Finally, a gate was drawn around the double-positive mWISP-1 antigen-specific population. All positive cells were sorted into reverse transcription mix chilled to 4°C at either 1 cell or 3 cells per well in a 96-well PCR plate.

### cDNA generation and transcriptionally active PCR (TAP)

Details of transcriptionally active PCR (TAP) including oligonucleotide and DNA fragment sequences are provided in published patent applications WO2010/097435 [34] and WO2010/097437 [35] and in Clargo *et al*. [9]. Briefly, cDNA from single B cells was prepared using Superscript III reverse transcriptase (Invitrogen) primed with oligo (dT). Antibody variable-region genes were then recovered via two rounds of PCR using either KOD DNA polymerase (EMD Millipore) or TaqPlus Precision DNA polymerase (Agilent) (mouse primary PCR). A primary PCR utilized gene-specific primers at both the 5’ and 3’ ends. The 5’ oligonucleotide set bound either at the 5’ end of the leader sequence (for rabbit variable regions) or at the 5’ end of the framework 1 region of the mature variable region sequence (for mouse variable regions). The 3’ reverse primer set annealed to CH1 or Cκ region respectively. In the secondary PCR, a single 5‘ forward oligonucleotide that annealed to a “tail” encoded at the 5’ end of the primary PCR product was used with a 3’ primer set that annealed in the J region. Not only did the secondary oligonucleotides introduce restriction sites to facilitate downstream cloning, but they also provided ~25 base-pair overlap regions; at the 5’ end with a human cytomegalovirus (HCMV) promoter fragment (plus a leader sequence for rat-derived fragments that were generated with the framework 1 primer set) and at the 3’ end with a heavy or light chain constant region fragment. Then, in a tertiary PCR, variable region DNA, HCMV promoter fragment and constant region fragment containing a poly-adenylation sequence were combined and amplified to produce two separate linear transcriptionally-active PCR (TAP) products, one encoding the heavy chain and the other the light chain. Rabbit variable regions were recombined with rabbit constant regions in the expression cassette whereas mouse variable regions were assembled with mouse constant regions to produce recombinant IgG molecules. PCR set up was performed using an Aviso Onyx liquid handling robot.

### Recombinant antibody expression

Heavy and light chain transcriptionally active PCR fragments were transiently co-transfected into Expi293 cells (Life technologies) using Expi293 fectamine (Life Technologies) as per manufacturers’ instructions. These were incubated for 7 days at 37°C in a 5% CO_2_ environment before the supernatants were harvested by centrifugation for further characterisation.

### IgG expression assessment

IgG levels in expi293 supernatants were assessed for both mouse and rabbit via ELISA. Briefly, goat anti-species Fc fragment-specific polyclonal antibodies (Jackson Immunoresearch) were coated onto 96 well ELISA plates at 2 μg/ml. Plates were blocked with 1% PEG_5000_ (Sigma) in PBS before four half-log serial dilutions of each transient expression sample were prepared and applied to the plates along with a dilution series of a purified rabbit or mouse IgG standard. Following washing, goat anti-species Fab fragment-specific antibodies conjugated to horseradish peroxidase (Jackson Immunoresearch) were added at a 1:5000 dilution. Plates were developed with 3, 3′, 5, 5′-Tetramethylbenzidine (TMB) (Sigma) before being read at 630nm on a synergy 2 microplate reader (BioTek). Sample concentrations were then calculated by interpolating sample values from the standard curves.

### Recombinant IgG antigen-binding assays

Binding of mouse-derived antibodies to target (IL-25 or TNFR2) was determined by ELISA. Briefly, 96-well ELISA plates were coated with 2 μg/ml of the target protein. Plates were blocked with 3% BSA (Life Technologies) in PBS. Recombinant TAP samples containing mouse IgG were added to the plates and finally the plates were revealed with goat anti-mouse Fc fragment-specific antibodies conjugated to horseradish peroxidase (Jackson Immunoresearch) at a 1:5000 dilution. Plates were developed with TMB before being read at 630nm on a Synergy 2 microplate reader (Bio Tek). Positive binders were determined based on the production of an absorbance value which was at least 4-fold higher than that produced by samples containing no antibody (background).

Binding of rabbit-derived antibodies was determined via homogenous fluorescence assay. Briefly, mouse WISP-1 (R & D systems) was biotinylated using Lightning link biotin (innova Bioscience) according to manufacturers’ instructions and coated onto Superavidin^TM^ beads (Bangs laboratories). Coated beads were then incubated with TAP supernatants containing recombinant rabbit IgG and anti-rabbit Fc-Alexa Fluor 647 (Jackson Immunoresearch) at a 1:5000 dilution for 1 hour. Plates were then read on an FMAT ABI 8200 (Applied Biosystems). Binders were determined as antibodies that showed a mean fluorescence signal greater than zero on WISP-1-coated beads and no signal on uncoated beads.

### Secondary PCR product sequencing and diversity assessment

To determine the sequence diversity from the sorted mouse B-cells, we performed DNA sequencing of the secondary PCR products. This was achieved using the secondary forward oligonucleotide for both the heavy and kappa chains as a primer. CDR3 sequences were extracted and used to assess diversity. Sequence diversity was assessed using a Principal Components Analysis (PCA) [[36]] similar to that described in Clargo *et al*. [9]. This provides a means to reduce the dimensionality of the data and generate an easy to interpret 2-dimensional data plot that illustrated the extent of diversity in our recombinant antibody panel. The relative position of the data points on the 2-D plot can be considered to be directly proportional to sequence identity across the data set based on the VH CDR3. Identical sequences resulted in the co-localization of data points on the 2-D plot (shown with multiple identifier flags) and related sequence families (those containing 80% sequence identity or higher in VH CDR3) have been identified either by using a single colour for the data points (IL-25Fig) or by collectively circling data points (TNFR2Fig). All other sequences were considered unique.

For the TNFR2 antibody panel, information regarding sequence uniqueness and functionality could be visualized on the same plot. TNF-blocking activity was indicated by shape type and colour of the data point (blue diamonds represent blocking antibodies, red squares represent non-blockers and black circles were antibodies where blocking data could not be reliably generated) and affinity was indicated by size of the data point (larger being of higher affinity).

### BIAcore analysis

Surface plasmon resonance was performed using a BIAcore T200 (GE Healthcare). All experiments were performed at 25°C. Goat F(ab’)2 anti-mouse Fcγ (Jackson Immunoresearch) was immobilised on a CM5 Sensor Chip (GE Healthcare) via amine coupling chemistry to a capture level of ~5000 response units. HBS-EP+ buffer (10 mM HEPES pH 7.4, 0.15 M NaCl, 3 mM EDTA, 0.05% (v/v) surfactant P20, (GE Healthcare) was used as the running buffer. A 10‒50 μl injection of each antibody sample (0.1‒5 μg/ml) was used to achieve ~100 RU of capture. Two 3 min injections of human TNFR2 (generated in-house) at 10 nM and 100nM were passed over the captured antibody. Affinity was estimated following a 15 min dissociation phase. To determine blocking activity, binding of TNFR2 was followed by a 1 min injection of human TNF (generated in house) at 150 nM, at a flow-rate of 30 μl/min. The surface was regenerated at a flow-rate of 10 μl/min by two 60 s injections of 40 mM HCl and a 30s injection of 5 mM NaOH. Double referenced background subtracted binding curves were analyzed using the T200 Evaluation software (version 1.0) following standard procedures. Kinetic parameters and estimated affinity values for most antibodies were determined from fitting a BIAcore algorithm using a single cycle kinetics 1:1 binding model except 4G3, 3C11, 3A9 and 3F7 which were fitted using a bivalent model and affinity estimates derived from k_a_1 and k_d_1.

## Results

### 1. Antigen-Specific Mouse Memory B-Cell Sorting

**Fig 2:**
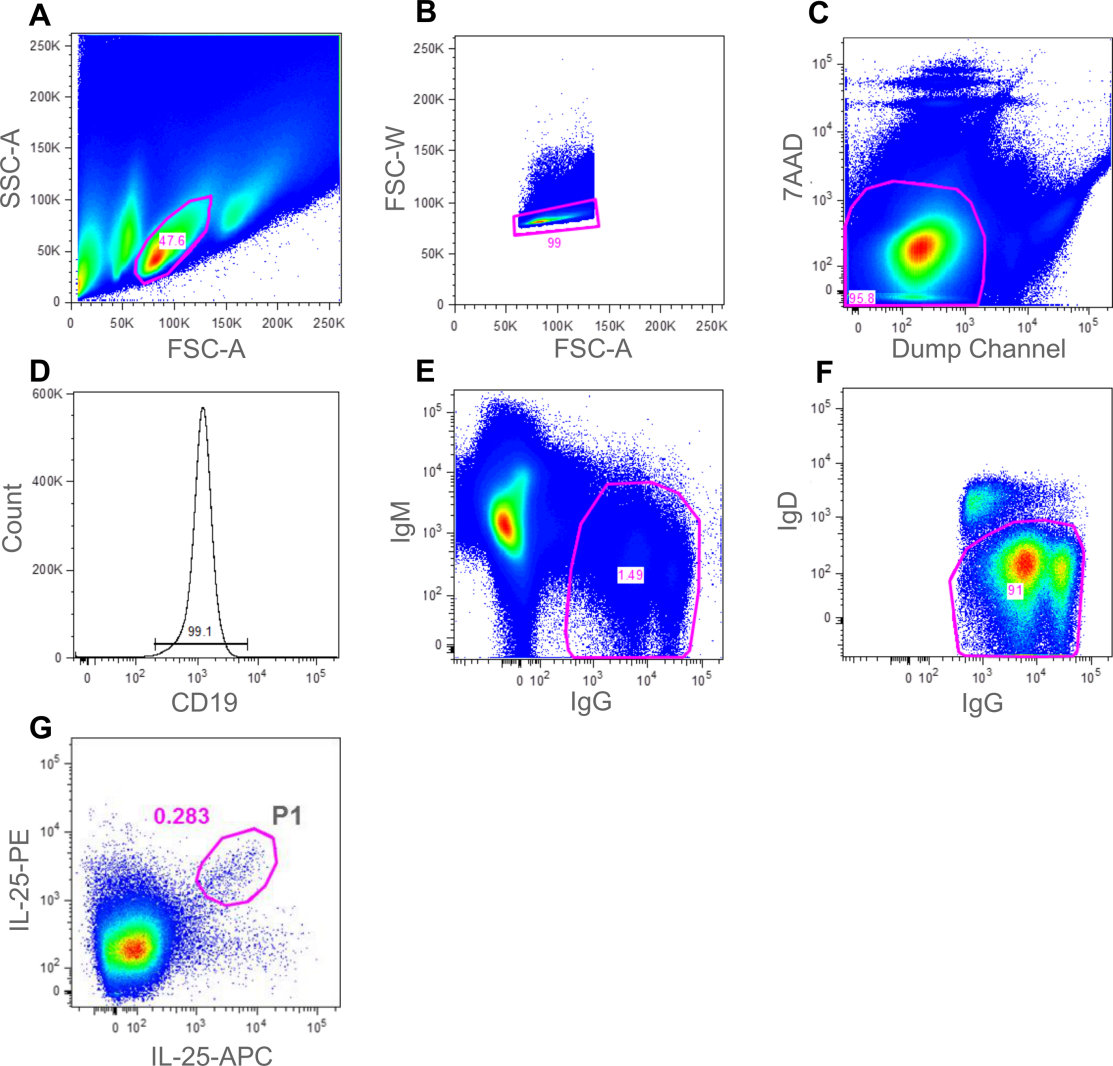
Gating strategy for the identification of antigen-specific mouse memory B cells from IL-25 immunised mice. Following cell enrichment using CD45R microbeads (Miltenyi Biotech), cells were analysed in a BD FACS ARIA III. A gate was drawn around the lymphocyte population (gated population represented 47.6% of events) (A). FSC-W and FSC-A were then used to eliminate doublets (gated population represented 99% of events) (B). T cells, macrophages, neutrophils and 7AAD^+^ dead cells were eliminated in the “dump channel” (gated population represented 95.8% of events) (C). CD19^+^ B cells were identified (gated population represented 99% of events) (D). IgG^+^/IgM^-^ B cells were then gated on (gated population represented 1.49% of events) (E). To further eliminate naïve B cells, IgD staining allowed gating for IgG^+^/IgD^-^ cells, (gated population represented 91% of events) (F). Finally, dual-colour antigen staining allowed a gate (P1) to be drawn around the double-positive population (gated population P1 represented 0.283% of events) (G). Single cells from gate P1 were sorted into a 96-well plate for single-cell RT-PCR.

**Fig 3:**
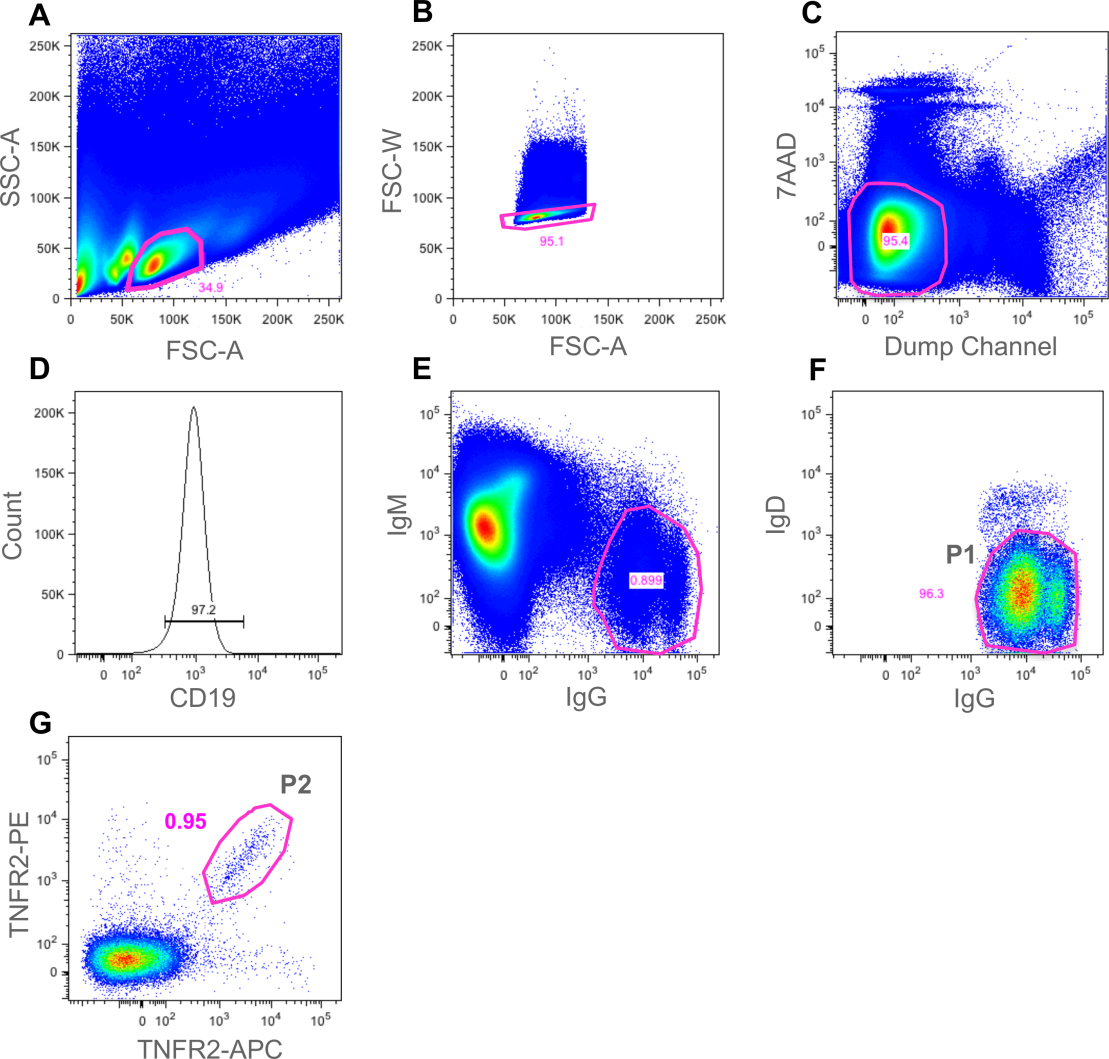
Gating strategy for identification of antigen-specific mouse memory B cells from TNFR2 immunised mice. Following cell enrichment using CD45R microbeads (Miltenyi Biotech), cells were analysed in a BD FACS ARIA III. A gate was drawn around the lymphocyte population (gated population represented 34.9% of events) (A). FSC-W and FSC-A were then used to eliminate doublets (gated population represented 95.1% of events) (B). T cells, macrophages, neutrophils and 7AAD^+^ dead cells were eliminated in the “dump channel” (gated population represented 95.4% of events) (C). CD19^+^ B cells were identified (gated population represented 97.2% of events) (D). IgG^+^/IgM^-^ B cells were then gated on (gated population represented 0.899% of events) (E). To further eliminate naïve B cells, IgD staining allowed gating for IgG^+^/IgD^-^ cells (gated population (gate P1) represented 96.3% of events) (F). In order to demonstrate the importance of the antigen staining step, we sorted single cells from the total IgG^+^/IgD^-^ cell population (gate P1). Finally, dual-colour antigen staining allowed a gate (P2) to be drawn around the double-positive antigen-specific population (gated population P2 represented 0.95%of events) (G). Single cells from gate P2 were sorted into a 96-well plate for single-cell RT-PCR.

Following immunisation, B cells were purified from splenocyte preparations using anti-B220 MACS. B cells were then stained using the antibody panel described in the materials and methods and summarised in Table 1. The panel of spectrally-distinct reagents was designed to maximise the ability to identify and sort human IL-25 or TNFR2-specific mouse memory B cells. The panel included the use of an anti-CD19 reagent, a positive B cell lineage marker, an anti-IgG monoclonal pool (specific to individual isotypes) to ensure staining of the class-switched population of memory B cells and negative stains for IgM and IgD to exclude naïve B cells. To increase the purity of the B cell subset further, a “dump channel” reagent set comprising anti-CD4/CD8, anti-GR1 and anti-F4/80 was included to eliminate T cells, neutrophils and macrophages respectively. We also included 7AAD to identify and remove dead cells. By including this extensive panel we were able to exclude contaminating cell types that may be able to bind antigen with low affinity (naïve B cells), those cell types that bind antigen non-specifically or those cells that masquerade as B cells (Bell and Gray, 2003). By using APC and PE conjugates, two intensely bright fluorophores, the antigen-specific B cell subset was extremely well-defined within the FACS software and a sorting gate could be easily drawn around this population prior to sorting. Figs 2 and 3 shows a number of FACS staining plots which ultimately lead to the identification of the IL-25-specific (Fig 2, gate P1) and TNFR2-specific (Fig 3, gate P2) IgG^+^ memory B cells. Fig 3, also highlights the total IgG^+^ memory B cell population (gate P1).

**Table 1:** Summary of antibody reagents used for identification of IgG^+^ antigen-specific mouse B cells.

Marker/Reagent	Cell type	Fluorophore
B220 (CD45R)	B-cells	MACS bead
CD19	B-cells	AF700
IgG	Memory B-cells	BV421
Antigen	Ag specific B-cell	PE
Antigen	Ag specific B-cell	APC
IgM	Naïve B-cells	PE-Cy7
IgD	Naïve B-cells	APC-Cy7
CD4	T-cells	FITC
CD8	T-cells	FITC
GR-1	Neutrophils	FITC
F4/80	Macrophages	FITC
7AAD	Dead cells	

An optimised panel of fluorophore-labelled reagents was developed to maximise the potential of identifying the IgG^+^ antigen-specific subset of B cells. Both B cell-specific and non-B cell specific (dump channel) reagents were employed.

The inclusion of antigen, labelled with two separate fluorophores, enabled us to sort those cells that were capable of binding both versions, thereby increasing the probability of identifying B cells that were highly specific to IL-25 or TNFR2.

For both the IL-25 and TNFR2 experiments, B cells from the double-positive antigen-specific population (Fig 2G, gate P1 (IL-25) and Fig 3G, gate P2 (TNFR2)) were sorted at single-cell per well into three 96-well plates containing reverse transcription reaction mix. To demonstrate the efficiency of the antigen staining step in the TNFR2 experiment, B cells from the total IgG^+^ memory B cell population (Fig 3F, gate P1) were sorted at single-cell per well into an additional 96-well plate. cDNA was generated from single cells and then heavy and light chain antibody variable region genes were amplified via two-stage PCR using gene-specific primers. In a third round of PCR variable region DNA products were combined with DNA fragments harbouring a promoter fragment and a mouse constant region fragment and amplified using pull-through primers to generate transcriptionally-active PCR (TAP) products for both the VH and VL. These PCR products were then used directly to transfect expi293 cells for the expression of antibody at 1 mL scale in 48-well blocks. Recombinant IgG-containing supernatant were harvested from cultures and then tested for binding against human IL-25 and human TNFR2 respectively via ELISA. The percentage of wells producing IgG and the percentage of those wells that demonstrated antigen-specificity is shown in Table 2 (IL-25) and Table 3 (TNFR2).

**Table 2:** Summary of recombinant IgG expression and antigen binding activity of antibodies derived from the IL-25-specific IgG^+^ B cell population.

Population	% IgG recoveries as TAP transients	% Antigen-specific IgG recovered
Antigen-specific memory B-cells (Fig 2G, gate P1)	53%	73%

Three 96-well plates containing single IL-25-specific memory B cells (sorted from gate P1, Fig 3G) were subjected to RT-PCR to recover variable region genes and generate transcriptionally-active PCR (TAP) products. Following generation of recombinant IgG from expi293 cells (Life Technologies), supernatants were tested by ELISA for the presence of IgG and for their ability to bind to recombinant human IL-25. Percentage of wells producing recombinant IgG and percentage of those demonstrating antigen binding is shown.

**Table 3:** Summary of recombinant IgG expression and antigen binding activity of antibodies derived from the total IgG^+^ population and the TNFR2-specific subset of cells.

Population	% IgG recoveries as TAP transients	% Antigen-specific IgG recovered
Total IgG^+^ memory B cells (Fig 3F, gate P1)	24%	0%
Antigen-specific memory B cells (Fig 3G, gate P2)	33%	79%

One 96-well plate containing single memory B cells from gate P1 (total IgG^+^ population) (Fig 3F) and three 96-well plates of single B cells from gate P2 (TNFR2-specific population) (Fig 3G) were subjected to RT-PCR to recover antibody variable region genes and generate transcriptionally-active PCR (TAP) products. Following generation of recombinant IgG from expi293 cells (Life Technologies), supernatants were tested by ELISA for the presence of IgG and for their ability to bind to recombinant human TNFR2. Percentage of wells producing recombinant IgG and percentage of those demonstrating antigen binding is shown.

**Fig 4:**
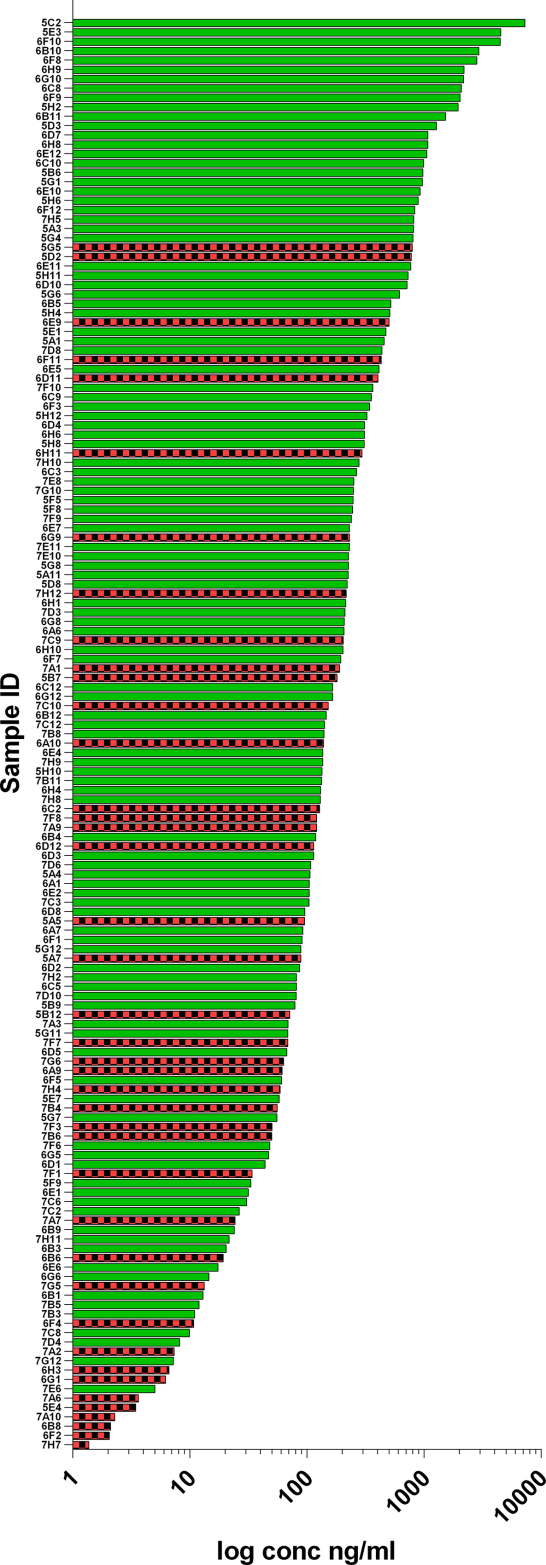
Recombinant IgG concentration range produced from IL-25 B cell sorting experiment. Heavy and light chain TAP fragments produced from single-sorted B cells were transiently co-transfected into Expi293 using ExpiFectamine (Life Technologies). After 7 days expression, supernatants were harvested and the concentration of mouse IgG measured using an sandwich ELISA with a purified mouse IgG standard. Data is shown for all wells that produced measurable antibody production. Green bars indicate those that were determined to bind IL-25 and red and black-check bars represent those which did not show binding to IL-25.

As shown in Table 2, transient expression of IgG from TAP products was successful from 53% of the sorted cells. Of these, 73% demonstrated binding to IL-25 via ELISA. In total, the IL-25 sorting experiment generated 112 IL-25-specific recombinant antibodies identified by ELISA. This represents an overall efficiency of 39% of sorted cells. Fig 4 illustrates the range of expression levels observed and highlights those wells that exhibited IL-25 binding (green bars). As can be seen, binding was observed in samples with a broad range of concentrations.

In addition to sorting the antigen-specific population, in the TNFR2 experiment we also sorted single cells from the total IgG^+^ memory B cell population (without gating on antigen). As shown in Table 3, the recovery and generation of recombinant IgG was similar for both the total IgG memory B cell gate (Fig 3F, gate P1) (24%) and the antigen-specific IgG^+^ memory B cells gate (Fig 3G, gate P2) (33%). However, antigen specificity was only recovered in the recombinant antibodies produced from gate P2 where the two-colour antigen stain was incorporated into the sorting parameters. This demonstrates the importance of a reliable and robust antigen-staining step when sorting from the diverse and extensive IgG^+^ memory B cell pool. A total of 75 hTNFR2-specific recombinant antibodies were identified by ELISA. This represented 79% of those wells that gave rise to recombinant IgG and an overall efficiency of 26% of sorted cells.

### 2. Characterisation of mouse antibodies

**Fig 5:**
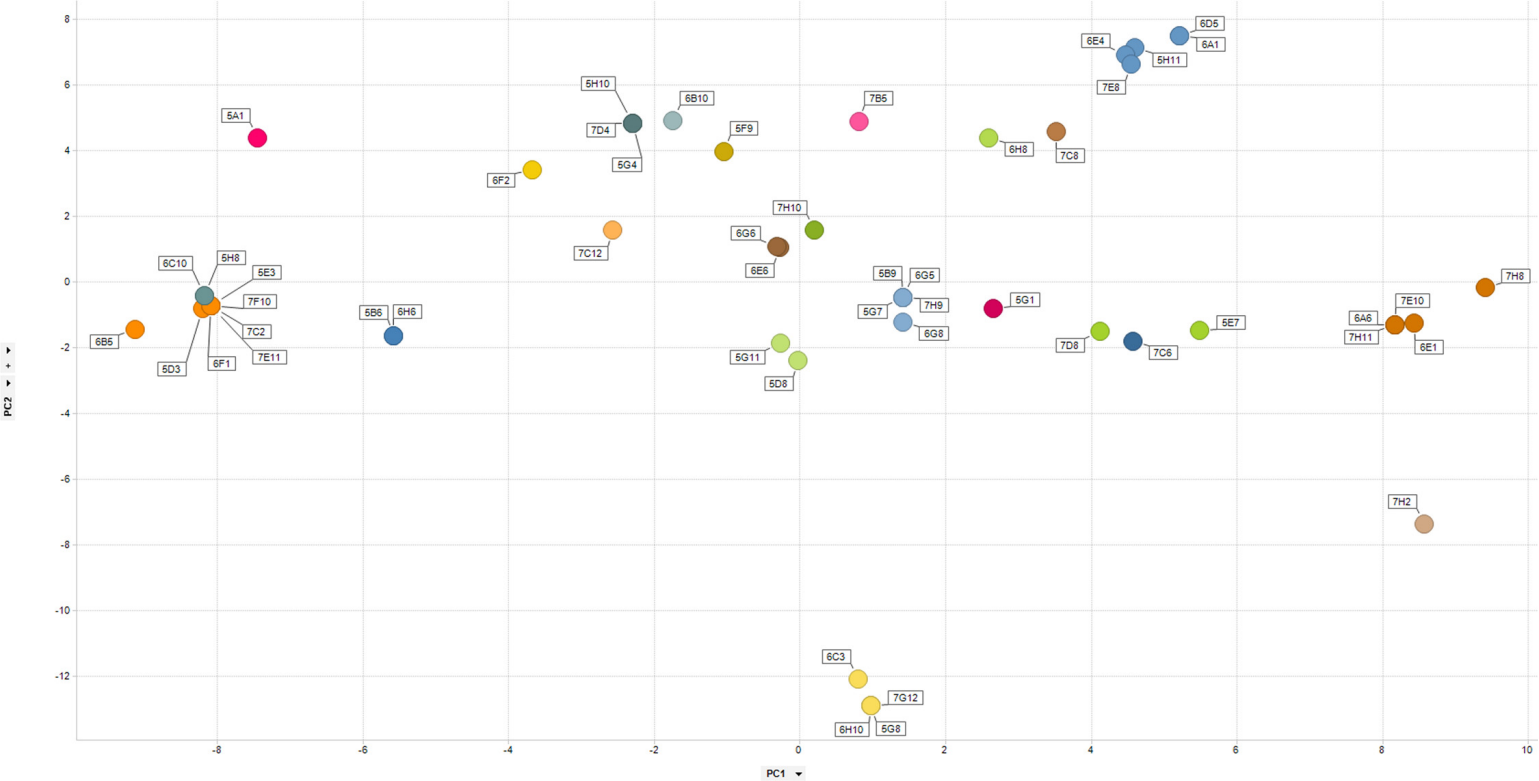
Diversity assessment of mouse anti-human IL-25 antibodies. For each antibody, VH CDR3 sequences were aligned in a pairwise manner to generate a sequence distance value. We performed a Principal Components Analysis (PCA)^34^ as a means to reduce down the dimensionality of this data and generate an easy to interpret 2-dimensional data plot that illustrated the extent of diversity in our recombinant antibody panel. Data for principle component (PC) 1 and 2 are shown on the X and Y axis respectively. Clustering analysis was performed and families of closely related sequences were assigned on the basis of sequence identity in the VH CDR3 regions of 80% or higher. Each separate antibody family has been represented by a unique colour.

In order to determine the diversity of the anti-IL-25 antibody panel, we performed DNA sequencing of the heavy chain variable region PCR products. This allowed us to rapidly identify CDR3 sequences for 50 of the IL-25-binding antibodies. Cloning and sequencing would likely have enabled the generation of full-length variable region sequences for all of the specific antibodies. However, the direct sequencing of the PCR product represented a straight forward and rapid way to approximate the diversity in the isolated antibody set and for this reason conventional cloning and subsequent sequencing was not pursued in this study. Despite only having data for a limited number of antibodies, significant diversity in our antibody panel was observed. Fig 5 illustrates a PCA analysis where the position of a data point within 2-dimensional space provides a measure of sequence similarity. Related sequences which constituted an antibody family were defined as those which shared a heavy chain CDR3 amino acid sequence containing 80% or higher sequence identity. Families are indicated through the use of a single colour on the PCA plot. We identified 23 unique families of antibody from the 50 analysed sequences. This demonstrates that the method described here is capable of producing highly diverse panels of recombinant antibody.

**Fig 6:**
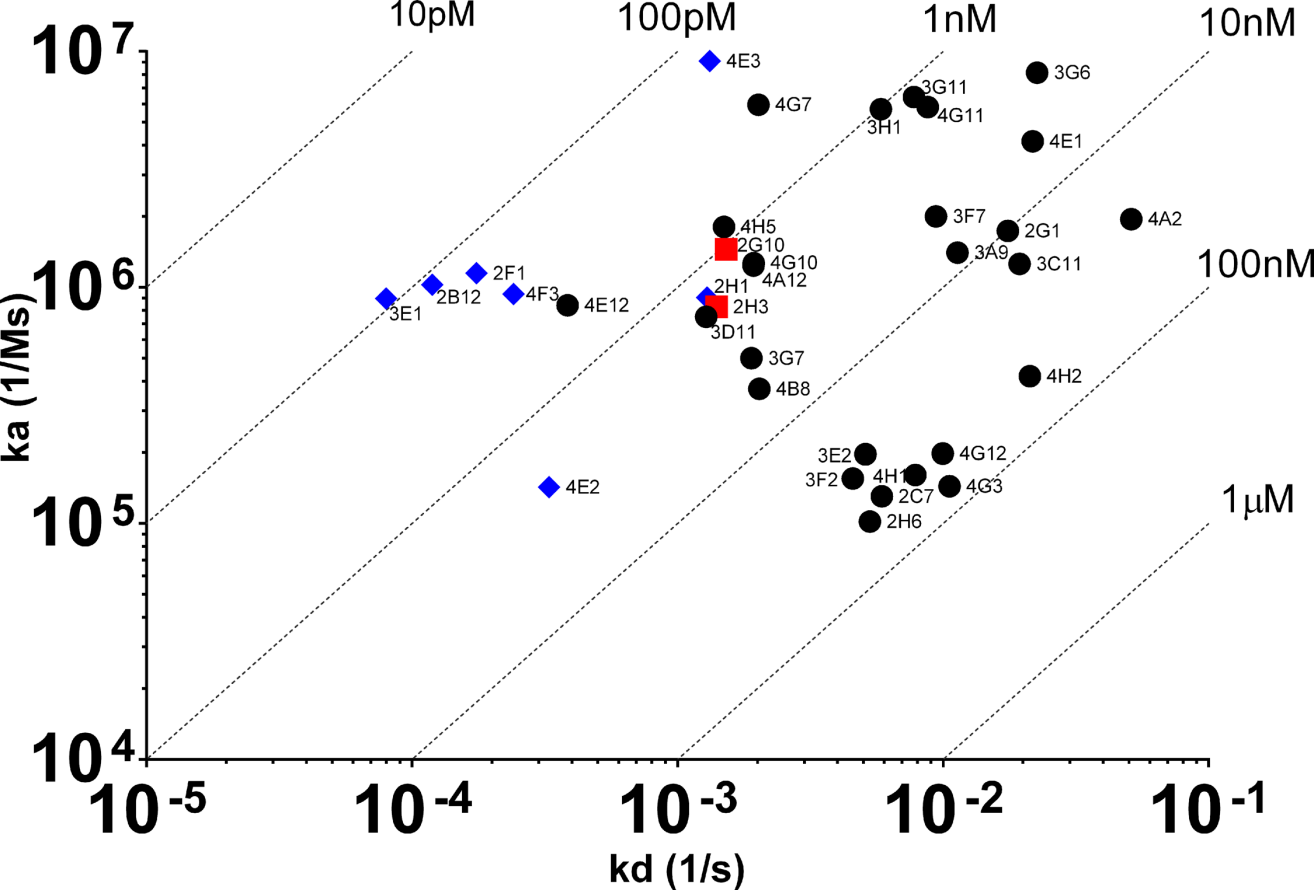
Affinity analysis of TAP-derived recombinant antibodies. Heavy and light chain TAP fragments were transiently co-transfected into Expi293 using ExpiFectamine (Life Technologies). After 7 days of expression, supernatants were harvested and the on-rate (k_a_), off-rate (k_d_) and affinity constant (K_D_) of mouse IgG against human TNFR2 was estimated on a BIAcore T200 applying a BIAcore 1:1 binding model except 4G3, 3C11, 3A9 and 3F7 which were fitted using a bivalent model and affinity estimates derived from k_a_1 and k_d_1. Blue diamonds represent blocking antibodies, red squares represent non-blockers and black circles were antibodies where blocking data could not be reliably generated.

We performed a similar diversity assessment with the recombinant antibodies produced from the TNFR2 sorting experiment. However, we were also interested in determining the quality of the recombinant antibodies. Therefore, following determination of antibody concentration in the expi293 supernatants (concentrations ranged between 80 ng/mL to 5000 ng/mL, data not shown), we performed a BIAcore experiment with the culture supernatants to gain some insight into the affinity of the antibodies. Only a subset of the antibodies which showed binding activity in the ELISA also showed a binding response in the BIAcore experiment (35 from 75 antibodies). The BIAcore experiment enabled us to determine estimates of affinity and K_D_ values are shown in Fig 6. As can be seen, an affinity range between 90 pM and 52 nM was estimated for this panel of anti-human TNFR2 antibodies. In order to determine if any of the antibodies could block the interaction of TNFR2 with its ligand, we included a human TNF binding step following the dissociation phase of the TNFR2 molecule in the BIAcore experiment. Blockers were identified as those antibodies which prevented the association of TNF with TNFR2. Fig 6 highlights those antibodies that were capable of blocking ligand binding (blue diamonds). Only those antibodies with a sufficiently high concentration and slow off-rate were capable of generating blocking data under the conditions used in this work. Cloning of variable regions and re-expression at a higher concentration would likely have allowed for a more complete blocking data set to have been generated. We successfully produced blocking data for nine recombinant antibodies. Of these nine antibodies, it was interesting to note that seven demonstrated the ability to prevent TNF binding to TNFR2 and the 4 highest affinity antibodies, including the 90 pM molecule, appeared to possess blocking activity. The data demonstrates that a single-B cell FACS approach can be employed for the generation of both blocking and non-blocking monoclonal antibodies with a range of affinities.

**Fig 7:**
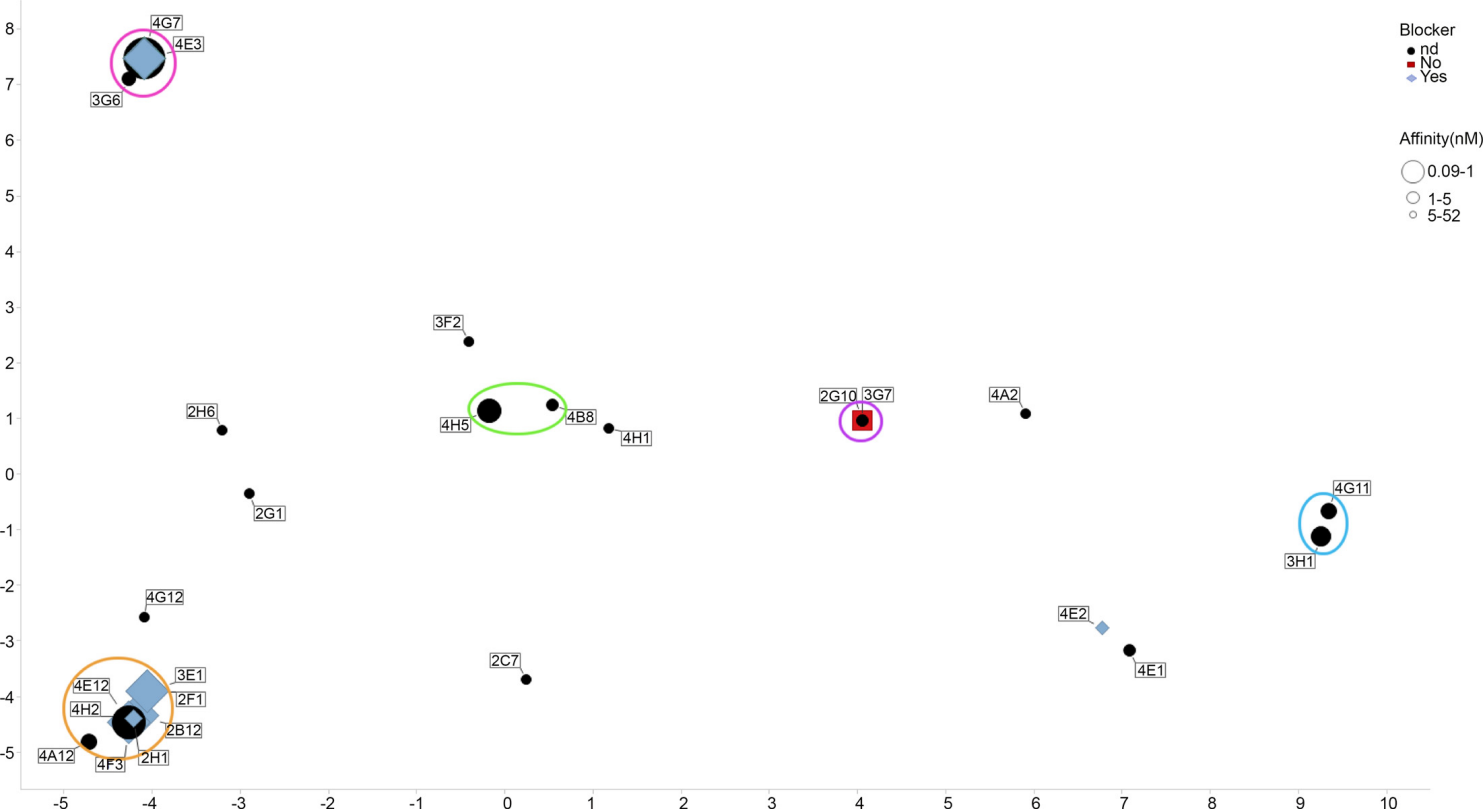
Diversity assessment of mouse anti-human TNFR2 antibodies. For each antibody, VH CDR3 sequences were aligned in a pairwise manner to generate a sequence distance value. We performed a Principal Components Analysis (PCA)^34^ as a means to reduce down the dimensionality of this data and generate an easy to interpret 2-dimensional data plot that illustrated the extent of diversity in our recombinant antibody panel. Data for principle component (PC) 1 and 2 are shown on the X and Y axis respectively. Clustering analysis was performed and families of closely related sequences were assigned on the basis of sequence identity in the VH CDR3 regions of 80% or higher. Clusters containing multiple sequences have been circled. All other sequences were considered unique. Identical sequences being co-located on the 2-D plot but indicated with multiple identifier flags. Blue diamonds represent blocking antibodies, red squares represent non-blockers and black circles were antibodies where blocking data could not be reliably generated. The size of the data point markers are indicative of affinity, with large markers representing high affinity (maximum of 90 pM) and smaller markers representing lower affinity (minimum of 52 nM).

As with the IL-25 sorting experiment we wanted to assess the diversity of the anti-TNFR2 antibody panel. We performed DNA sequencing on the amplified heavy chain variable region gene PCR fragments of the antibodies which had exhibited binding in the Biacore experiment. In order to visualise the data, we performed a PCA which also provided an overview of the affinity range and blocking activity of the antibodies. Fig 7 shows the CDR3 sequence analysis of the VH genes from 26 antibodies. As noted previously, cloning of variable region genes would likely have facilitated the generation of the complete sequencing dataset for all 35 antibodies. However, despite the lack of data for some antibodies, including one blocker and one non-blocker, we were still able to make an assessment of diversity in our recombinant antibody panel. As can be seen, based on having a VH CDR3 sequence identity of 80% or higher, 14 unique families of antibody were identified. Analysis of the blocking antibodies suggested the presence of 2 distinct families of antibody. As expected, the confirmed non-blocking antibody that we successfully recovered sequence data for, appeared to lie in a completely distinct cluster to the blocking antibodies.

Both the IL-25 and TNFR2 experiments demonstrate that the FACS method described here represents a robust and reliable method capable of generating panels of highly diverse and functional antibodies from mice within a short time frame.

### 3. Antigen-specific rabbit memory B-cell Sorting

Antibodies specific for rabbit B cell and other immune cell markers are less readily available and for this reason we employed a smaller panel of reagents to identify the antigen-specific subset of B cells. Due to the lack of reagents, we were also unable to apply a pre-enrichment step using B cell-specific beads (e.g. MACS) prior to flow cytometry. In this case, PBMCs from rabbits immunised with mouse WISP-1 were used as a source of B cells. PBMCs were stained using the antibody panel described in the materials and methods and summarised in Table 4. As with the mouse B cell experiment, we employed an anti-IgG stain to identify the class-switched memory B cell subset which were capable of expressing IgG. However, we were unable to introduce a positive B cell surface marker stain due to lack of availability of reagents. To facilitate the rabbit sorting experiment we included an anti-IgM stain to identify naïve B cells and a dump channel consisting of a number of anti-T cell reagents. We also included 7AAD to enable identification and removal of dead cells.

**Table 4:** Summary of antibody reagents used for identification of IgG^+^ antigen-specific rabbit B cells.

Marker/Reagent	Cell type	Fluorophore
IgG	Memory B-cells	PE
Antigen	Ag specific B-cell	Alexa-Fluor 488
Antigen	Ag specific B-cell	Alexa-Fluor 647
IgM	Naïve B-cells	PE-Cy7
CD4	T-cells	PerCP
CD8	T-cells	PerCP
Pan-T cell	T cells	PerCP
7AAD	Dead cells	

An optimised panel of fluorophore-labelled reagents was developed to maximise the potential of identifying the IgG^+^ antigen-specific subset of B cells in rabbit PBMC samples. Both B cell-specific and non-B cell specific (dump channel) reagents were employed.

**Fig 8:**
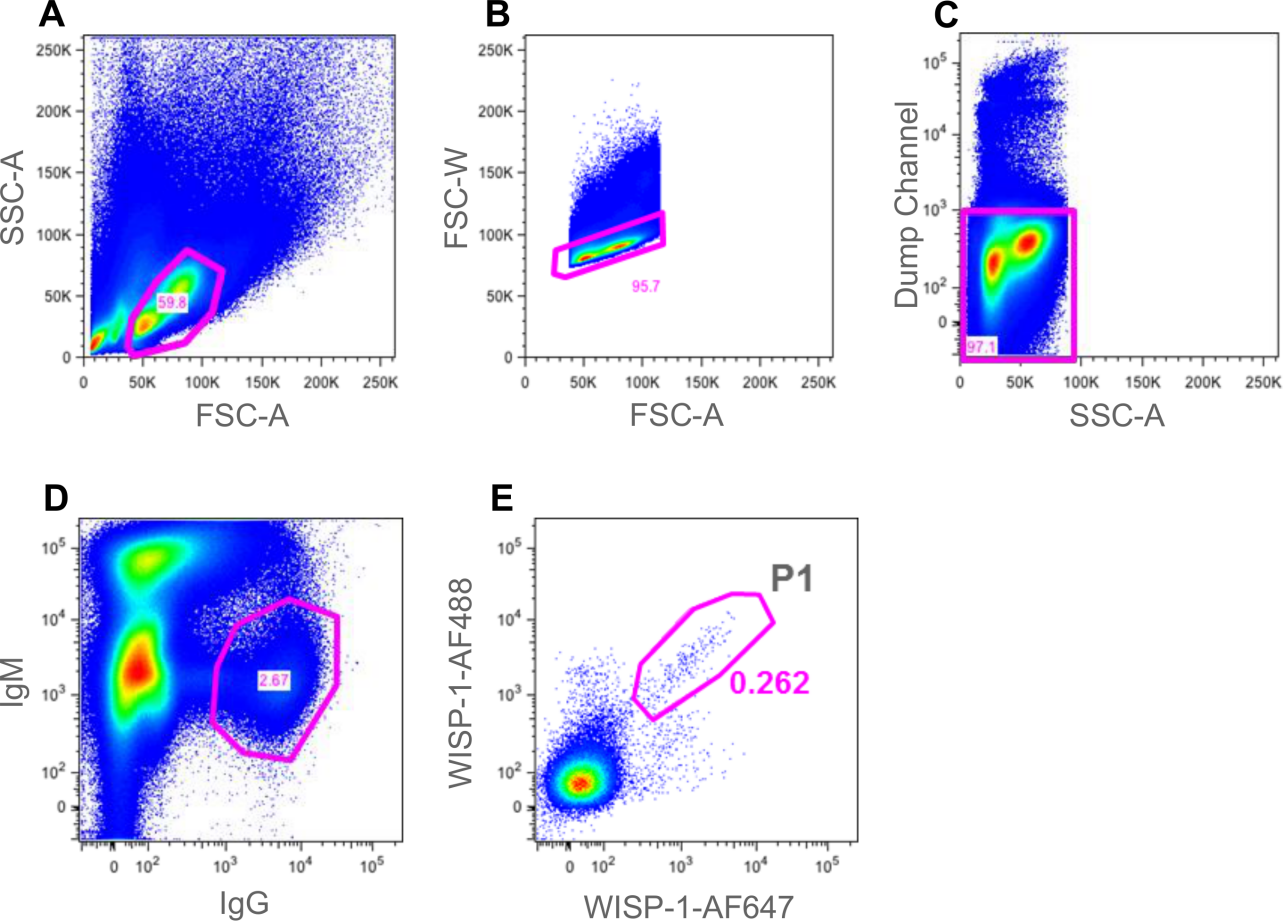
Gating strategy for identification of antigen-specific rabbit memory B cells. Cells were analysed in a BD FACS ARIA III. A gate was drawn around the lymphocyte population (gated population represented 59.8% of events) (A). FSC-W and FSC-A were then used to eliminate doublets (gated population represented 95.7% of events) (B). 7AAD^+^ dead cells and T cells were eliminated in the “dump channel” (gated population represented 97.1% of events) (C). IgG^+^/ IgM^-^ B cells were identified and gated on (gated population represented 2.67% of events) (D). Finally, a gate (P1) was drawn around the double-positive mWISP-1 antigen-specific population (gated population represented 0.262% of events) (E). Cells from gate P1 were sorted into a 96-well PCR plate at either one or three cells per well for subsequent RT-PCR.

Following staining, cells were assessed using a BD FACS Aria III multi parameter flow cytometer. FACS plots showing the cell staining data and subset identification are shown in Fig 8. Antigen-specific IgG^+^/IgM^-^ B cells were identified (see Fig 8, gate P1) and sorted at either one or three cells per well of a 96-well PCR plate containing reverse transcription reaction mix (44 wells at both one and three cells per well, 88 wells in total). We included a three-cell per well sort as we reasoned that due to the lack of reagents for detecting both specific positive and negative B cell markers, the observed antigen-reactive population may be less pure than that identified in the well-defined mouse system, i.e. that some contaminating non-B cells may be present in the P1 gate (Fig 8). Having performed the sort, cDNA generation and PCR was carried out as described above with the mouse experiment. TAP was performed using rabbit constant regions generating expression cassettes capable of producing rabbit IgG following transfection of Expi293 cells.

The resulting recombinant antibodies, present in culture supernatant, were then screened for binding to mouse WISP-1 using a homogeneous fluorescence-based binding assay where biotinylated WISP-1 protein was immobilised on Superavidin^Tm^ beads. Table 5 summarises the percentage of wells that yielded IgG following TAP product transfection and expression and the percentage of those wells that went onto demonstrate antigen binding in an ELISA.

**Table 5:** Summary of recombinant IgG expression and binding recoveries.

Population	Cells per well sorted	% IgG recoveries as TAP transients	% WISP-1-specific IgG recovered
Antigen-specific memory B-cells (gate P1)	1	0%	0%
Antigen-specific memory B-cells (gate P1)	3	18%	75%

One 96-well plate containing 44 wells filled with single B cells and 44 wells filled with three B cells from gate P1 (as shown in Fig 8) were subjected to RT-PCR to recover antibody variable region genes and generate transcriptionally-active PCR (TAP) products. Following generation of recombinant rabbit IgG from HEK-293 cells, supernatants were tested by ELISA for the presence of IgG and for their ability to bind to mouse WISP-1 in a homogeneous fluorescence-based FMAT binding assay.

As can be seen, recombinant IgG expression recovery was lower than that observed for mouse and we only observed IgG production and antigen binding in wells that received 3 cells prior to RT-PCR. The heavy and light chain variable region gene recovery was significantly lower than we typically achieve with single-cell RT-PCR from rabbit B cells [9]. Despite this, of the 8 recombinant antibodies that were successfully generated, 6 demonstrated WISP-1 binding. The data suggests that not all cells in the P1 gate are of B cell lineage but of those which are, a large percentage appear to be antigen-specific. Further characterisation of these antibodies was not undertaken.

## Discussion

A number of single B cell technologies that allow the efficient sampling of the B cell repertoire of an immunized animal have emerged in recent years [8]. These technologies have provided significant advantages over traditional hybridoma approaches. Circumventing the inefficient fusion event which is required to produce a hybridoma means that, in principle, an entire B cell repertoire can be sampled using single B cell technologies. This ultimately equates to more efficient identification of rare B cells producing antibodies with favourable profiles. Also, because these methods are not reliant on a myeloma fusion partner, they can be applied to multiple species, including humans, where reagents allow for identification and isolation of the single B cells. This provides more flexibility with regard to which animal species is used for producing antibodies and results in larger and more diverse panels of antibody from which to select molecules with desirable characteristics. This is particularly advantageous in therapeutic antibody development where the criteria for success are extensive. The technique described here, particularly when used in conjunction with complementary technologies sampling other B cell subsets, facilitates the generation of multiple “starting points” from which amino acid sequences can be engineered and humanized to produce therapeutic candidates. These candidates can then be screened for activity, stability, expression, specificity, epitope targeting etc before selecting a lead molecule with the most attractive profile for clinical development.

In this study, we describe the use of a multiparameter flow cytometry method to isolate naturally-occurring, antigen-specific IgG^+^ memory B cells, directly from the spleen and blood of immunized mice and rabbits respectively. Staining of both B cells and non-B cells allowed for highly pure populations of antigen-specific B cells to be identified. The use of antigen, labelled with two spectrally-distinct fluorophores, allowed for sensitive identification of those cells capable of dual-binding the target molecule with high specificity rather than cells binding non-specifically to one of the protein-fluorophore conjugates. In both Figs 2 and 3 (mouse) and Fig 8 (rabbit), antigen-staining on a single colour can be observed and so the dual-antigen stain helped to enrich the true positive population. A similar concept was described by Townsend *et al*. [[29]]. However, they used the technique for analysis of cell populations only whereas we have extended the approach, employing a broader panel of fluorescent reagents, to identify and sort individual antigen-specific B cells for subsequent recombinant monoclonal antibody generation.

The mouse system is relatively well furnished with a range of antibody reagents that enable both positive identification of B cells and the staining and elimination of non-B cell subsets such as T cells, neutrophils and macrophages which are likely to be present in the same immune tissue samples that are the source of B cells. Importantly, Bell and Gray [37] demonstrated that so-called “antigen-capturing cells” of non-B cell lineage exist in mice. These cells, which include macrophages, monocytes, neutrophils and dendritic cells, are able to capture circulating IgG through FcγRI molecules, which if the antigen-specific IgG component is at a high enough concentration, can subsequently result in capture of antigen onto the cell surface. By incorporating both positive and negative cell stains into the mouse protocol, we were able to exclude the non-B lymphocyte antigen-capturing cells allowing for the accurate identification of the B cell subset. Combining this with a dual-antigen labelling technique allowed us to identify and sort a highly enriched population of antigen-specific B cells. Following RT-PCR, generation of TAP products and expression in a transient expi293 system, we were able to demonstrate recovery of 112 anti-human IL-25 and 75 anti-human TNFR2 recombinant antibodies. This represented an overall efficiency of 40% and 26% of sorted cells respectively within the antigen-specific gate (Fig 2, gate P1 for IL-25 and Fig 3, gate P2 for TNFR2). This compares very favourably to other reports of antigen-specific cell sorting. For example, Weitkamp *et al*. [30] attempted to sort rotavirus (RV)-specific B cells from human blood using fluorescent virus-like particles as a source of target. They showed that 23% of sorted cells showed antigen binding following *in vitro* activation to induce antibody secretion but following RT-PCR, the efficiency dropped to 1.3% of the sorted population. Smith *et al*. [7] employed a sorting approach to sample the transient plasmablast population without an antigen labelling step. Despite being able to achieve up to 22% recovery of recombinant antigen-specific antibodies from single-sorted cells, the frequency of antigen-specific plasmablasts can be significantly lower than the 70% reported in this paper. Frequencies of 10% or lower may be observed and in such circumstances, the efficiency would drop to 2–3%.

In order to determine the quality of antibodies generated from the mouse memory B cell sorting experiment, we attempted to estimate affinity of the anti-TNFR2 panel via SPR using transiently-expressed antibody. Thirty five recombinant antibodies generated data that could be processed using the BIAcore analysis software to produce an estimated K_D_. It is likely that poor affinity (slow on-rate, fast off-rate) combined with relatively low expression levels accounted for those antibodies which failed to show binding by BIAcore but did by ELISA. Of those antibodies where kinetics could be determined, an affinity range between 90 pM and 52 nM was observed. This confirmed that the FACS technique employed here is capable of sampling the memory B cell repertoire efficiently, enabling the generation of antibodies with a broad range of affinities, i.e. not limited to antibodies with high affinity or with high surface-B cell receptor expression that may have been preferentially labelled with antigen. Although we gated on the entire antigen-specific population in this experiment it may be possible to selectively sort the high affinity subset by taking those cells that exhibit a high antigen-specific to total IgG staining ratio [38,39]. However, high affinity does not always correlate with antibody function hence this type of approach should be treated with caution.

In order to determine the neutralisation capability of the recombinant anti-human TNFR2 antibodies, we included a human TNF binding step in the BIAcore experiment to identify those antibodies that bound at the ligand-binding site of the receptor and prevented TNF association. The assay was applicable to those antibodies which possessed a slow off-rate where a stable antibody-TNFR2 complex could be maintained on the surface of the BIAcore sensor. At the antibody concentrations produced in this work (<10 μg/ml), reliable blocking data could not be obtained for those antibodies with a relatively fast off-rate (k_d_ >2 x 10^−3^). It should be noted however that the BIAcore experiment utilised here was primarily designed to determine binding kinetics. An alternative assay design would likely have generated more extensive blocking data but this was considered to be beyond the scope of the existing study.

Nine antibodies successfully produced ligand association data with seven that demonstrated the ability to prevent TNF binding to TNFR2. Interestingly, the four highest affinity antibodies in the recombinant panel appeared to possess blocking activity. The data demonstrates that a single-B cell FACS approach can be employed for the generation of both high affinity blocking and non-blocking monoclonal antibodies. It may also be possible to identify blocking antibodies directly in the FACS experiment through the introduction of a labelled binding partner for the target and identifying those B cells that do not positively stain for this. For example, in the TNFR2 experiment described here the introduction of human TNF labelled with a spectrally-distinct fluorophore may have allowed us to identify TNFR2^+^/TNF^-^ memory B cells presumably producing antibodies that can block TNF binding to the receptor. However, this additional parameter was not tested in the present study.

In order to assess diversity in the recombinant mouse antibody panels for both the IL-25 and TNFR2, we performed sequencing of the variable regions using the amplified secondary PCR products. A principal components analysis (PCA) was performed allowing diversity to be illustrated on a 2-dimensional plot. Based on having a VH CDR3 sequence identity of 80% or higher, 23 and 14 unique families of IL-25- and TNFR2-specific antibodies were identified from a total of 51 and 26 sequenced antibodies respectively. This shows that the technique described here is robust and capable of generating large panels of diverse antibodies from two separate immunisation experiments representing both a soluble cytokine target (IL-25) and an extracellular domain of a cell-surface receptor (TNFR2). The broad application of this technique to multiple target classes will facilitate its widespread use in the antibody discovery field.

For the TNFR2 analysis we were able to build in both affinity and blocking activity into the PCA visualisation figure. These features of the antibodies were displayed by the size and colour of the individual data point markers respectively (Fig 7). As described above, blocking data was only obtained for the higher affinity antibodies with slower off-rates (k_d_<2 x 10^−3^). Analysis of the blocking antibodies revealed the presence of 2 distinct families of antibody. Both families encompassed variants where blocking data was not successfully produced presumably due to insufficient affinity. For example, one of the blocking families included three antibodies 4E3, 4G7 and 3G6, which all possessed very high on-rates (k_a_), but only 4E3, the highest affinity (slowest off-rate) variant in this family, was confirmed as a blocker with both 3G6 and 4G7 failing to provide sufficiently reliable data to determine blocking activity. Based on the similarity of the VH CDR3, it is likely that all three antibodies are capable of binding to a very similar epitope on TNFR2 and exhibit blocking activity but due to the faster off-rates of 3G6 and 4G7, this was not possible to confirm experimentally. As expected, the confirmed non-blocking antibody that we successfully recovered sequence data for (2G10), appeared to cluster separately to the blocking antibodies and possessed a very different variable region sequence.

The diversity assessments over two separate sorting experiments with different antigens suggested that the memory B cell repertoire of the mouse represents an excellent subset from which to derive highly diverse panels of high quality antibody that target different epitopes (at least two confirmed based on blocking data generated for the TNFR2 antibodies; blocking and non-blocking). Having access to a number of different V region sequences provides the potential to select those antibodies with favourable functional and biophysical characteristics. In the case of therapeutic antibody development, having multiple sequence options provides an increased chance of being able to successfully humanise a rodent sequence and transition the molecule into a clinical development program.

We wanted to extend the concepts applied in the mouse sorting experiment to the rabbit system. Rabbits offer an excellent source of high quality antibodies and provide the opportunity to generate anti-mouse orthologue research reagents and species cross-reactive antibodies, e.g. anti-mouse and anti-human cross-reactive [9,40]. Although reagents for identifying B cell subsets and other cell types in the rabbit are limited, we wanted to perform a proof of concept experiment to demonstrate that recombinant antigen-specific rabbit monoclonal antibodies could be generated using FACS. Reagents to eliminate T cells, naïve IgM^+^ B cells and dead cells were incorporated along with a positive anti-IgG stain and a dual-antigen labelling step similar to that used with the mouse. In this case we used the antigen mouse WNT1-inducible-signaling pathway protein 1 (WISP-1), a molecule implicated in tumour pathogenesis and fibrosis. Following labelling of PBMCs from WISP-1-immunised rabbits, B cells were sorted at either one or three cells per well of a PCR plate. As expected, the lack of other staining reagents, especially in the dump channel, meant that efficiency was reduced compared to the mouse. We were unsuccessful in recovering heavy and light chain variable region gene pairs from wells that received single cells. However, we were able to generate paired VH and VL and recombinant IgG from wells that received 3 cells. The paired variable region recovery rate was relatively low (18% of wells). Typically we obtain cognate pairs from approximately 75% of isolated single rabbit B cells [9]. This suggests that in the FACS approach described here that not all identified cells within gate P1 (Fig 8) were of B cell lineage. Alternatively, the rabbit memory B cells may possess relatively low levels of mRNA or be more susceptible to template degradation following cell sorting resulting in lower variable region recovery compared to mouse. Without a broader set of antibody reagents to characterise the identified rabbit cell population, it is difficult to determine the exact cause of the lower recovery rates. It is also unclear why those wells that received single cells failed to produce paired variable region genes given that the recovery rate with 3 cells per well was 18%, i.e. expected rate of recovery should have been 6% (approx. 2–3 wells). However, in those wells where VH and VL genes were recovered and recombinant IgG generated from TAP products, 75% (6 monoclonal antibodies) also exhibited binding to the target antigen WISP-1 in a homogeneous fluorescence-based binding assay. We did not characterise these antibodies any further. This relatively high percentage of antigen-reactive antibodies in the recombinant IgG panel suggested that a highly enriched specific population of B cells was correctly identified using the staining protocol described and that further refinement of the reagent panel through addition of antibodies for labelling monocytes and neutrophils for example, may allow for elimination of contaminating, non-B cell populations. Such additional reagents are not readily available from commercial sources. Therefore, bespoke antibody discovery campaigns may be required to provide these antibodies.

We have demonstrated through the use of multi-parameter flow cytometric sorting that the IgG^+^ memory B cell repertoire of mice and rabbits represent an excellent source from which to produce recombinant monoclonal antibodies. When combined with transcriptionally-active PCR (TAP), recombinant IgG (or other antibody fragment formats) can be efficiently produced within the space of one week. Alternative techniques for interrogating the memory B cell repertoire have been described previously, including the use of *in vitro* activation and screening [[41,42]]. Although, these culturing approaches have the advantage of being able to screen antibodies for various activities and functions prior to antibody gene cloning, the FACS-based approach described here provides an attractive alternative and offers the possibility of being able to screen large numbers of recombinant antibodies produced from a transient HEK-293-based (or other mammalian cell) system where antibody expression may be higher and in some situations provide improved compatibility with cell-based functional screening assays. The cell culturing approaches do however provide the option to screen against cell-surface molecules that are difficult to obtain as soluble and purified proteins. Such targets, which include GPCRs and ion channels, would be far more challenging to prosecute using the FACS approach described in this study. It may be possible to employ peptides that map to extracellular regions but it is common for these to adopt non-native conformations. Alternatively, fluorescently-tagged membrane preparations, virus-like particles or thermo-stabilised mutant receptor proteins could be employed [30,43,44].

In summary, the generation of large and diverse panels of high quality monoclonal antibodies to therapeutically-relevant target molecules is reliant on the use of a range of discovery technologies that allow for efficient interrogation of the antibody repertoire. The FACS approach described here represents a complementary technology to established antibody discovery methods and provides an additional mechanism by which to identify fit-for-purpose antibodies to an ever expanding array of targets.
